# An optimized VMAT planning technique for hippocampal‐sparing whole‐brain radiotherapy

**DOI:** 10.1002/acm2.70518

**Published:** 2026-02-17

**Authors:** Bei Liu, An Liu, Terence Williams, Ji Hyun Kim

**Affiliations:** ^1^ Division of Radiation Oncology City of Hope National Medical Center Duarte California USA

**Keywords:** hippocampus sparing, VMAT whole brain plan

## Abstract

**Purpose:**

Whole brain radiation therapy (WBRT) has been shown to provide palliation but with negative neurocognitive effects associated with radiation‐induced damage to the hippocampus. Sparing of the hippocampus has been shown to reduce the risk of neurocognitive deficit. The goal of this study is to develop a VMAT planning strategy for WBRT with hippocampal avoidance (HA) that will minimize hippocampal dose while achieving homogeneous coverage of whole brain PTV.

**Materials and methods:**

A retrospective study was performed on 10 patients previously treated for intracranial lesions. CT and MRI fused images were used to delineate the whole brain and hippocampus. Strictly following RTOG 0933 atlas guidelines, the hippocampus was manually delineated by a single radiation oncologist. A 4‐arc noncoplanar VMAT approach was studied and compared with a 2‐arc coplanar method for a prescription of 30 Gy in 10 fractions. We first generated an intermediate plan that optimized only the portion of the whole‐brain PTV located farther from the hippocampus. Optimization was then continued by adding the remaining portions of the whole‐brain PTV to the optimization structure. In the 4‐arc VMAT optimization, a similar intermediate plan was utilized but in addition to this, the hippocampus was blocked for both entry and exit. Dosimetric parameters from both techniques were compared by paired *t*‐test.

**Results:**

When compared to RTOG 0933 dosimetric criteria, both the 2‐arc coplanar plans and 4‐arc noncoplanar met them with a great margin. However, the 4‐arc noncoplanar VMAT approach achieved dramatically better dose sparing for hippocampus as measured by D_0.03cc_ (10.55 Gy vs 12.66 Gy, *p* < 0.001), and D_mean_ (8.43 Gy vs 9.74 Gy, *p* < 0.001) when compared to the 2‐arc coplanar plans. Dose homogeneity of whole brain PTV was also improved substantially in the 4‐arc noncoplanar approach as measured by D_2%_ (32.49 Gy vs 32.90 Gy, *p* < 0.001), D_98%_ (28.55 Gy vs 27.97 Gy, *p* < 0.001), and the homogeneity index (HI) (0.126 vs 0.155, *p* < 0.001).

**Conclusion:**

Using an intermediate plan in VMAT optimization, both the 2‐arc coplanar plans and 4‐arc noncoplanar plans meet RTOG 0933 criteria with a great margin, while the 4‐arc noncoplanar VMAT planning method for HA‐WBRT further improves the hippocampus sparing, as well as whole brain PTV coverage and dose homogeneity.

## 1 INTRODUCTION

About 20% of cancer patients will develop brain metastases,^1^, ^2^ however, the likelihood of developing brain metastasis can be much higher in certain cancer types such as lung, breast, melanoma, and renal cell carcinoma.^3^, ^4^, ^5^ With the advancements in treatments with improved systemic control and growing use of sensitive magnetic resonance imaging (MRI) there has been an increasing incidence of brain metastasis.^6^ Each year, 70,000‐400,000 new cases of brain metastases are diagnosed in the US.^7^ Whole brain radiotherapy (WBRT) is commonly administered for brain metastases to alleviate symptoms, provide intracranial disease control, and reduce risk of death due to neurologic causes.^8^, ^9^

Besides brain metastases, WBRT is also commonly used for patients with small‐cell lung cancer, to prevent the spread of cancer cells from lungs to brain.^10^, ^11^, ^12^ However, WBRT comes with the risk of neurocognitive decline. The hippocampus plays a crucial role in human memory and learning because it contains neural stem cells responsible for memory function. In efforts to preserve cognitive function, hippocampus avoidance (HA) WBRT was proposed and developed.

Using nine‐field IMRT beam arrangement in combination with 7 or 9 couch kicks, clinical trial RTOG 0933 demonstrated that uniform dose coverage can be achieved to the whole brain PTV to a prescription dose of 30 Gy in 10 fractions while maintaining decent hippocampus dose sparing to prevent neurocognitive functional decline.^13^ Whole brain PTV was defined as the volume of whole brain subtracting a 5 mm margin expansion around the hippocampus. Per RTOG 0933 protocol planning goals and dose constraints were as follows: whole brain PTV D_2%_ ≤ 37.5 Gy and D_98%_ ≥ 25 Gy; bilateral hippocampus D_100%_ ≤ 9 Gy and D_max_ ≤ 16 Gy, optic nerve D_max_ and optic chiasm D_max_ ≤ 37.5 Gy.

While the conventional bilateral‐field photon beam technique was often used to make a simple treatment plan and easy treatment delivery, HA WBRT has become the standard of care for patients with good performance status and without metastasis in the HA region for those who receive WBRT for brain metastasis following RTOG 0933 and NRG CC001.^14^ Treatment guidelines have more recently incorporated HA WBRT for the treatment of brain metastasis.^15^

Volumetric modulated arc therapy (VMAT) is becoming more and more widely adopted to achieve homogeneous whole brain dose distribution with hippocampus sparing and to reduce neurotoxicity caused by radiation. The dose constraints for the hippocampus in RTOG 0933 were mainly driven by feasibility in treatment planning.^6^ Several studies have shown sparing the hippocampus in HA‐WBRT improved cognitive function preservation after HA‐WBRT.^13^, ^14^, ^15^, ^16^, ^17^ After the publication of RTOG 0933 and NRG CC001, there has become an increase in utilization of HA‐WBRT for brain metastases, and researchers have proposed various treatment planning methods to improve whole brain PTV coverage and hippocampus sparing in HA‐WBRT such as coplanar, noncoplanar, HyperArc, and RapidArc plans.^18^, ^19^, ^20^, ^21^, ^22^, ^23^ In this work, we proposed a 4‐arcs noncoplanar method, which uses an intermediate plan while blocking the hippocampus in the optimization, to achieve improvements in both hippocampal sparing and whole brain PTV dose coverage. We compared this method with a 2‐arcs coplanar method, which is a common planning practice for many institutions including ours for HA‐WBRT, but still used our intermediate plan in optimization for both planning methods.

## 2 METHODS AND MATERIALS

### 2.1 Patient selection and target delineation

Retrospective WBRT with hippocampal sparing using 2 arc coplanar and 4 arc noncoplanar VMAT technique were done on 10 randomly chosen patients previously treated for intracranial lesions. High resolution MRI and CT simulation images of each patient were fused. The hippocampus for each patient was delineated following the RTOG 0933 protocol.

### 2.2 Treatment planning

**FIGURE 1:**
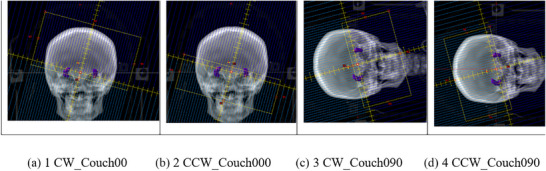
(a) 1 CW_Couch00 1 (b) 2 CCW_Couch000 1 (c) 3 CW_Couch090 1 (d) 4 CCW_Couch090 These four figures show the DRR of each arc at gantry angle 179°.

Treatment planning was performed in Eclipse V16 using Millennium 120 MLC model and 6 MV photons on an IEC scale Varian 21ix LINAC which does not support jaw‐tracking feature. The isocenter was placed at the center of the left and right hippocampus. In the 2‐arc coplanar VMAT planning, arc 1 CW_Couch000 and arc 2 CCW_Couch000 are two coplanar arcs with gantry angles range from 181° to 179°. Arc 1 is clockwise (CW) treating the superior part of brain and arc 2 is counterclockwise (CCW) treating the inferior part of brain, with 2 cm overlap between these two arcs (Figure 1a,b). In addition to the two coplanar arcs described above, the 4‐arc noncoplanar VMAT plans used two noncoplanar arcs at couch angle 90°: arc 3 CW_Couch090 and arc 4 CCW_Couch090 both with a collimator angle of 15° (Figure 1c–d). The gantry angles of both noncoplanar arcs ranged from 0° to 179°.

**FIGURE 2:**
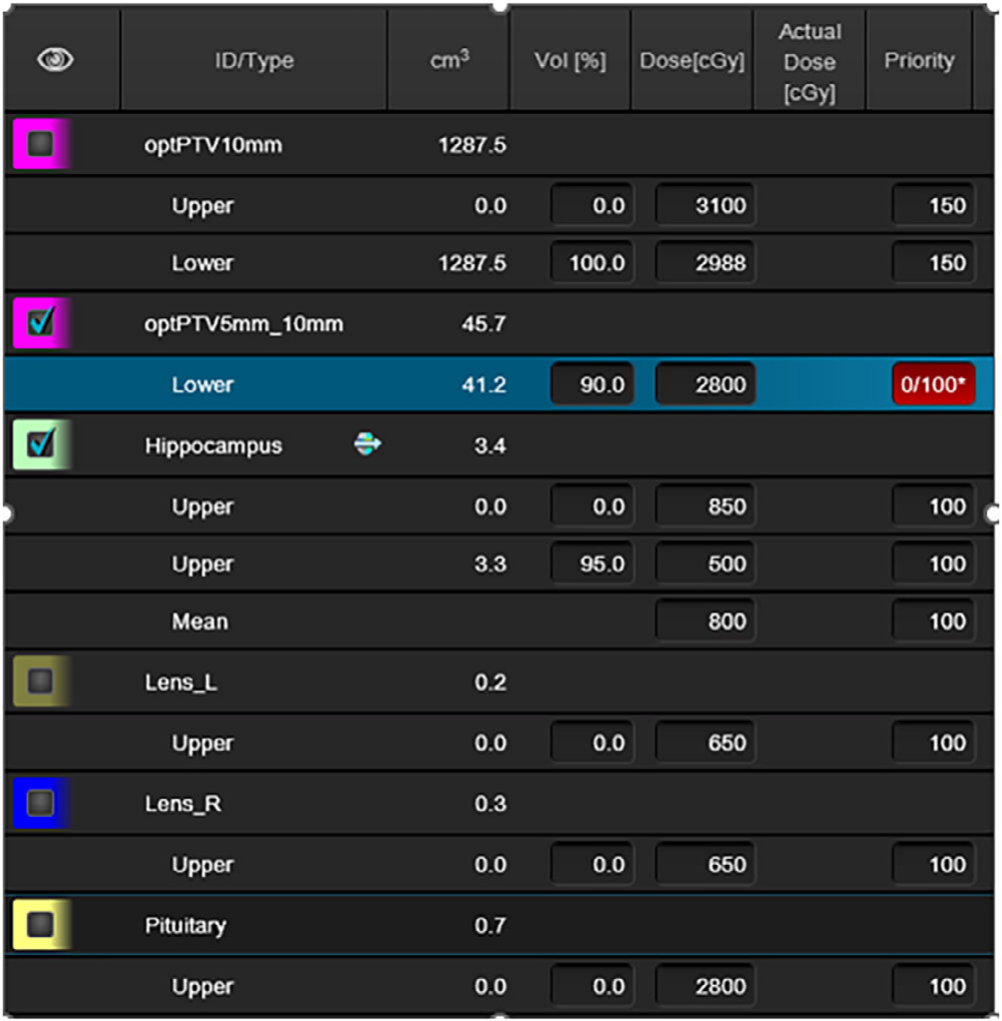
Optimization objectives used for 2‐arc and 4‐arc VMAT plans. Hippocampus was blocked using entry + exit in the 4‐arc VMAT optimization, but not in the 2‐arc VMAT optimization. * For the constraint of optPTV5mm_10 mm, priority 0 was used for the intermediate plan, and priority 100 was used for “continue the previous optimization”.

The dose calculation algorithm is Acuros 16.1 with 0.25 cm resolution, and PO 16.1 optimizer is used for optimization. Two optimization PTVs were created: optPTV10mm was created by subtracting a 10 mm expansion around hippocampus from the whole brain, and optPTV5mm_10 mm was created by subtracting optPTV10mm from whole brain PTV. The plan optimization consists of 2 steps. The first step was to create an intermediate plan optimized for optPTV10mm using optimization objectives in Figure 2 by setting the priority of optPTV5mm_10 mm as 0, with automatic NTO priority as 100. In the second step, we optimized the intermediate plan by choosing “continue the previous optimization” instead of “start again” and setting the priority of optPTV5mm_10 mm as 100. We continued the optimization by going back to MR Level2. In the 4‐arc VMAT optimization, in addition to the previous steps, the hippocampus was blocked for both entry and exit. The goal of the optimization is to minimize hippocampus dose while achieving homogeneous dose coverage to the whole brain PTV, and to ensure both right and left lens doses are less than 7 Gy and pituitary dose less than 30 Gy, in accordance with our institutional dose constraints. We did not set any optimization objectives for optical chiasm and optical nerves, because their RTOG 0933 maximum dose constraints of ≤ 37.5 Gy is inherently satisfied when using these optimization strategies.

### 2.3 Treatment plan evaluation

All plans were normalized so that V_30Gy_ of the whole brain PTV was 95%, and DVH indices for the whole brain PTV and OARs were compared. Besides the dose indices D_2%_ and D_98%_ of whole brain PTV, the dose homogeneity index (HI) was calculated using the definition in International Commission on Radiation Units and Measurements (ICRU) Report 8:3^24^

(1) $$\mathrm{HI}=\frac{D_{2\%}-D_{98\%}}{D_{50\%}}\;$$

A lower HI value means better PTV dose homogeneity. For hippocampus dose evaluation, the mean dose, minimum dose D_100%_ and maximum dose D_0.03cc_ were compared.

Portal dosimetry was used for IMRT QA for each plan. As the American Association of Physicists (AAPM) in Medicine Task Group 218 and 307 recommended, 3% dose difference and 2 mm distance‐to‐agreement (DTA) were used in gamma analysis.^25^, ^26^ Beam‐on time for each plan, which was the summation of the time from each arc's beam‐on to its beam‐off, was also recorded while performing IMRT QA, Bean‐on time was compared between the 4‐arc noncoplanar and the 2‐arc coplanar methods, along with the total monitor unit (MU) comparison.

### 2.4 Statistical analysis

R (Ver. 4.2.2) statistical package was used to compare the hippocampus dose indices and PTV coverage indices between the 4‐arc noncoplanar plans and 2‐arc coplanar plans, using paired *t*‐test. A *p*‐value less than 0.05 indicates the difference is statistically significant.

## 3 RESULTS

### 3.1 PTV dose coverage and hippocampus sparing

**FIGURE 3:**
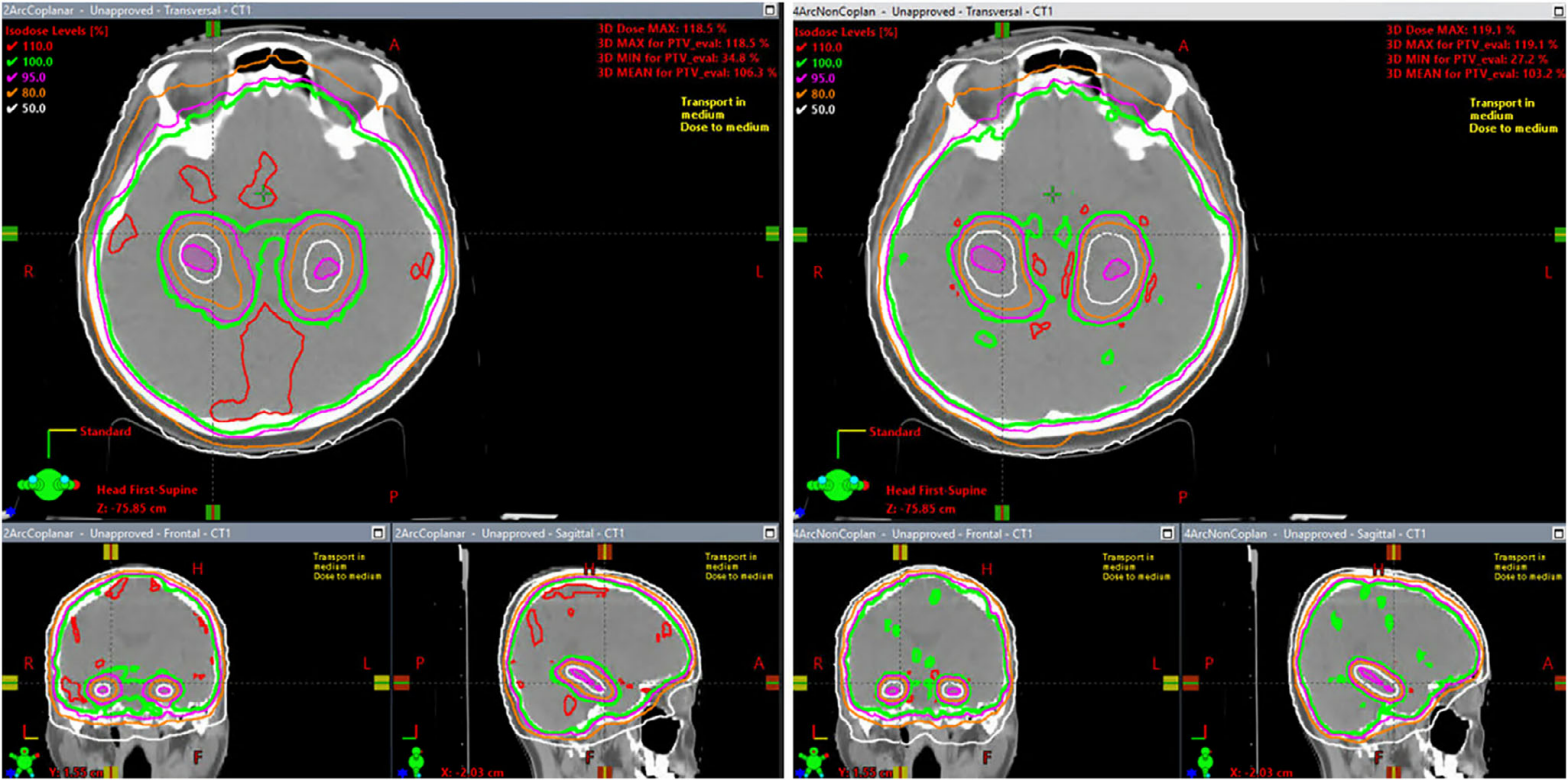
Side‐by‐side plan evaluation comparison between the isodose lines of a 2‐arc coplanar plan and a 4‐arc noncoplanar plan for an HA‐WBRT patient (2‐arc coplanar plan on the left and 4‐arc noncoplanar plan on the right).

The goal of this work is to find a VMAT treatment planning strategy of HA‐WBRT to minimize hippocampus dose, while maintaining dose coverage and homogeneity to whole brain PTV. Figure 3 shows a side‐by‐side plan evaluation comparison between the isodose lines of a 2‐arc coplanar plan and a 4‐arc noncoplanar plan for an HA‐WBRT patient. The magenta color wash structure is the hippocampus. This figure shows that: (1) dose sparing of hippocampus is clearly better in the 4‐arc noncoplanar plan, as seen from the 50% white isodose lines; (2) the dose coverage for the whole brain PTV in‐between the bilateral hippocampus was also substantially better in the 4‐arc noncoplanar plan, as illustrated by the 100% green isodose line in the axial and coronal view; (3) there is substantially less brain volume receive dose higher than 110% of prescription dose in the 4 arc noncoplanar plan, as seen from the 110% red isodose lines. Therefore, the 4‐arc noncoplanar plan achieved superior hippocampus sparing as well as whole brain PTV coverage and dose homogeneity.

**FIGURE 4:**
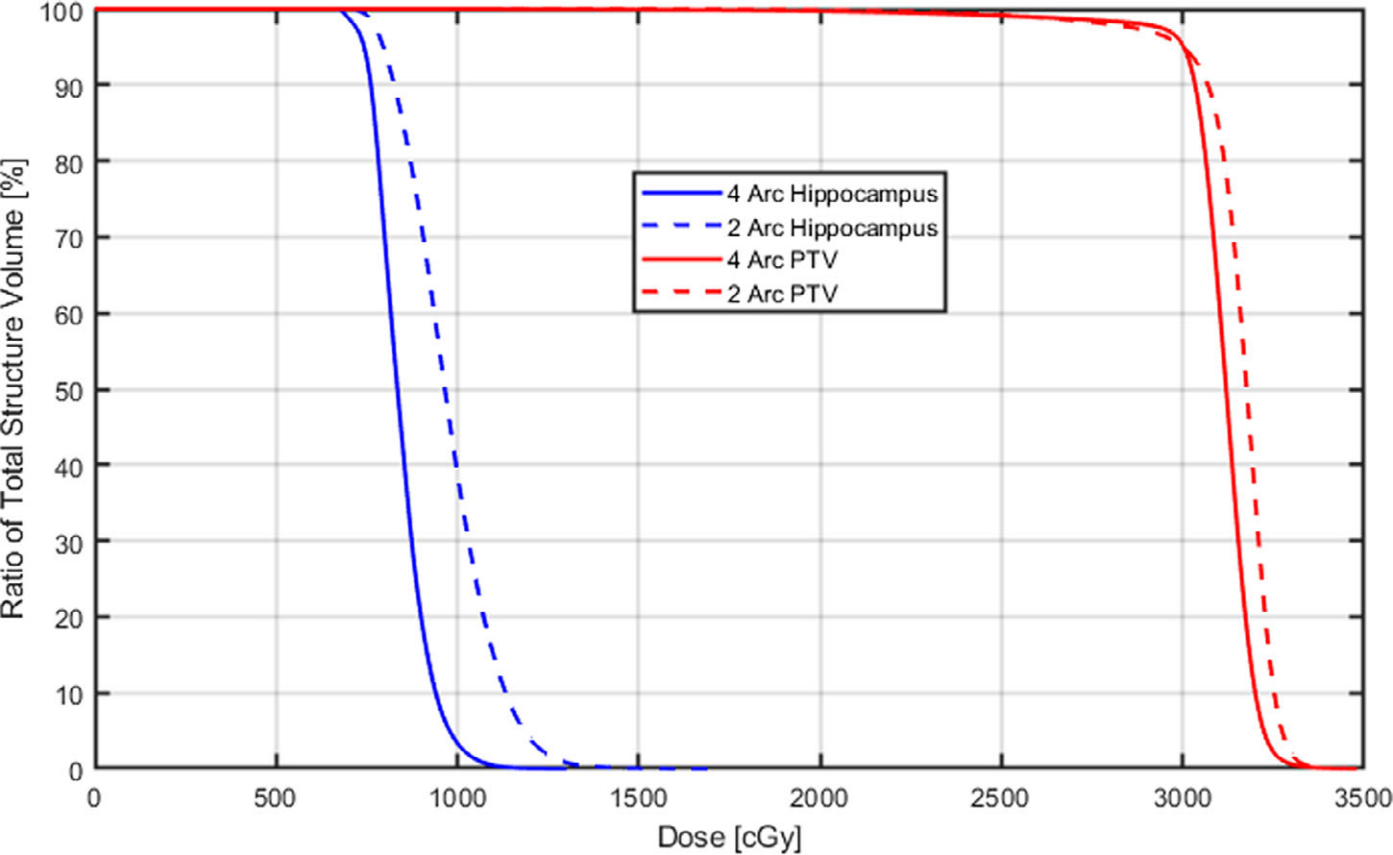
Mean DVH comparison of whole brain PTV and hippocampus between 2‐arc coplanar and 4‐arc noncoplanar methods of all 10 patients.

All the HA‐WBRT plans, both the 2‐arc coplanar or the 4‐arc noncoplanar VMAT plans met the RTOG 0933 and NRG CC001 PTV coverage requirement and OAR constraints, as well as our institution's OAR tolerance for pituitary and lens. Mean dose volume histograms (DVH) for whole brain PTV and hippocampus were constructed by combining the DVH data from all 10 patients. Figure 4 compares the mean DVHs between the plans made by 2‐arc coplanar and the 4‐arc noncoplanar methods, for whole brain PTV and hippocampus. The 4‐arc noncoplanar plans achieved improvements in hippocampus sparing and HI for whole brain PTV coverage.

Comparisons of the mean dosimetric indices and their standard deviation (mean ± SD) of all 10 patients for whole brain PTV, hippocampus, and other OARs between the 4‐arc noncoplanar VMAT plans and the 2‐arc coplanar VMAT plans are shown in Table 1. D_0.03cc_ was used to evaluate all the OAR's max point dose constraints.^27^ Whole brain PTV dose coverage and dose sparing on hippocampus are well within the RTOG 0933 tolerance, while the 4‐arc noncoplanar VMAT plans achieved dramatically better dose sparing for hippocampus as measured by D_0.03cc_ and D_mean_: average D_0.03cc_ are 10.55 Gy vs 12.66 Gy, a 16.7% dose reduction; and average D_mean_ are 8.43 Gy vs 9.74 Gy, a 13.4% dose reduction, with paired *t*‐test *P*‐value less than 0.001 for both D_0.03cc_ and D_mean_. The 2.3% dose reduction of D_100%_: 7.16 Gy vs 7.33 Gy for hippocampus is not as prominent as D_0.03cc_ and D_mean_ even though it is still statistically significant with a *p*‐value of 0.04. For other OARs such as pituitary, lens, optic nerves, and chiasm, they all met their constraints, respectively, in either the 2‐arc coplanar or 4‐arc noncoplanar method. We did not calculate the *P*‐value since our goal is not to minimize the dose of the OARs other than hippocampus, as long as their doses met constraints. Dose homogeneity of whole brain PTV was also improved substantially in the 4‐arc noncoplanar approach when compared to 2 arc coplanar: D_2%_ are 32.49 Gy vs 32.90 Gy, and D_98%_ are 28.55 Gy vs 27.97 Gy, respectively; the homogeneity indices (HI) are 0.126 vs 0.155.

**TABLE 1:** Comparison of the dosimetric indices (mean ± SD) of whole brain PTV, hippocampus, and other OARs between 4‐arc noncoplanar VMAT plans and 2‐arc coplanar VMAT plans. The coverage of PTVs of all plans was normalized to V_30Gy_ = 95%.

Structures	Dosimetric indices	Constraints	2‐arc VMAT coplanar	4‐arc VMAT noncoplanar	*P*‐value
Whole brain PTV	D_2%_ (Gy)	<37.5 Gy (RTOG0933)	32.90 ± 0.19	32.49 ± 0.15	<0.001
	D_98%_ (Gy)	>25 Gy (RTOG0933)	27.97 ± 0.44	28.55 ± 0.35	<0.001
	HI	NA	0.155 ± 0.016	0.126 ± 0.009	<0.001
Hippocampus	D_100%_ (Gy)	<9 Gy (RTOG0933)	7.33 ± 0.27	7.16 ± 0.21	0.04
	D_0.03cc_ (Gy)	<16 Gy (RTOG0933)	12.66 ± 0.55	10.55 ± 0.22	<0.001
	D_mean_ (Gy)	NA	9.74 ± 0.44	8.43 ± 0.26	<0.001
Pituitary	D_0.03cc_ (Gy)	<30 Gy (COH)	29.07 ± 0.35	28.24 ± 0.56	
Right len	D_0.03cc_ (Gy)	<7 Gy (COH)	6.73 ± 0.20	6.61 ± 0.30	
Left len	D_0.03cc_ (Gy)	<7 Gy (COH)	6.78 ± 0.13	6.66 ± 0.32	
Right optic nerve	D_0.03cc_ (Gy)	<37.5 Gy (RTOG0933)	31.01 ± 18.02	29.13 ± 12.51	
Left optic nerve	D_0.03cc_ (Gy)	<37.5 Gy (RTOG0933)	31.26 ± 2.20	28.68 ± 1.88	
Optic chiasm	D_0.03cc_ (Gy)	<37.5 Gy (RTOG0933)	33.35 ± 0.69	32.38 ± 0.64	

### 3.2 Dose of organ at risk (OAR) other than the hippocampus

As seen from Table 1, the OAR doses of all our plans, both 2‐arc coplanar plans and 4‐arc noncoplanar plans met their constraints. Among them, the optic nerves and optic chiasm met their RTOG0933 constraints with a large margin without adding any specific objectives in the VMAT optimization process, since all the plans have decent dose homogeneity. Pituitary and lens met their institutional constraints by adding corresponding objectives in VMAT optimization.

### 3.3 MU, beam‐on time and IMRT QA

Comparison of the mean MU and mean beam‐on time between 2‐arc coplanar plans and 4‐arc noncoplanar plans, where the beam‐on time of a plan is the sum of the time from each arc's beam on to its beam off, is shown in Table 2. The average MU of 4‐arc noncoplanar plans is 213%, more than double of the average MU of 2‐arc coplanar plans, indicating substantially more modulation in the 4‐arc VMAT plans. However, the beam‐on time of the 4‐arc noncoplanar plans is only about 165% of the beam‐on time of the 2‐arc coplanar plans on average, which is because the beam‐on time is mainly controlled by the gantry rotation time for low dose rate arcs: a 2‐arc plan contains two full arcs, while a 4‐arc plan contains two full arcs and two 180° partial arcs. Counting 30 seconds of couch rotation time and 10 seconds of beam mode‐up time of the two noncoplanar arcs, the average beam delivery time of a 4‐arc noncoplanar plan is 270.7 seconds, close to double the average delivery time of a 2‐arc coplanar plan. Portal dosimetry IMRT QA for all the 2‐arc plans and 4‐arc plans are excellent, with an average passing rate of 100% and 99.7%, respectively.

**TABLE 2:** Average MU, beam‐on time and IMRT QA passing rate of gamma analysis for 2‐arc coplanar plans and 4‐arc noncoplanar plans.

	2‐arc VMAT coplanar average ± SD	4‐arc VMAT noncoplanar average ± SD
MU	841.2 ± 33.4	1790.4 ± 193.5
Beam‐on time (sec)	140.2 ± 0.8	230.7 ± 13.0
Passing rate of gamma (%)	100 ± 0.0	99.7 ± 0.17

## 4 DISCUSSIONS

In the VMAT optimization, we started from an easy task by creating an intermediate plan using optPTV10mm as PTV. This intermediate plan achieves excellent hippocampus sparing and optPTV10mm coverage easily. Then we continued the optimization based on the intermediate plan and started from MR Level2 by adding optPTV5mm_10 mm as optimization PTV to improve the coverage of the whole brain PTV. This method achieved a favorable local minimum of the cost function that spares the hippocampus without compromising coverage of whole brain PTV. The effectiveness of this method is supported by the fact that both 2‐arc coplanar plans and 4‐arc noncoplanar plans using this method met the RTOG 0933 tolerance by a considerable margin in both hippocampus sparing and whole brain PTV coverage.

**FIGURE 5:**
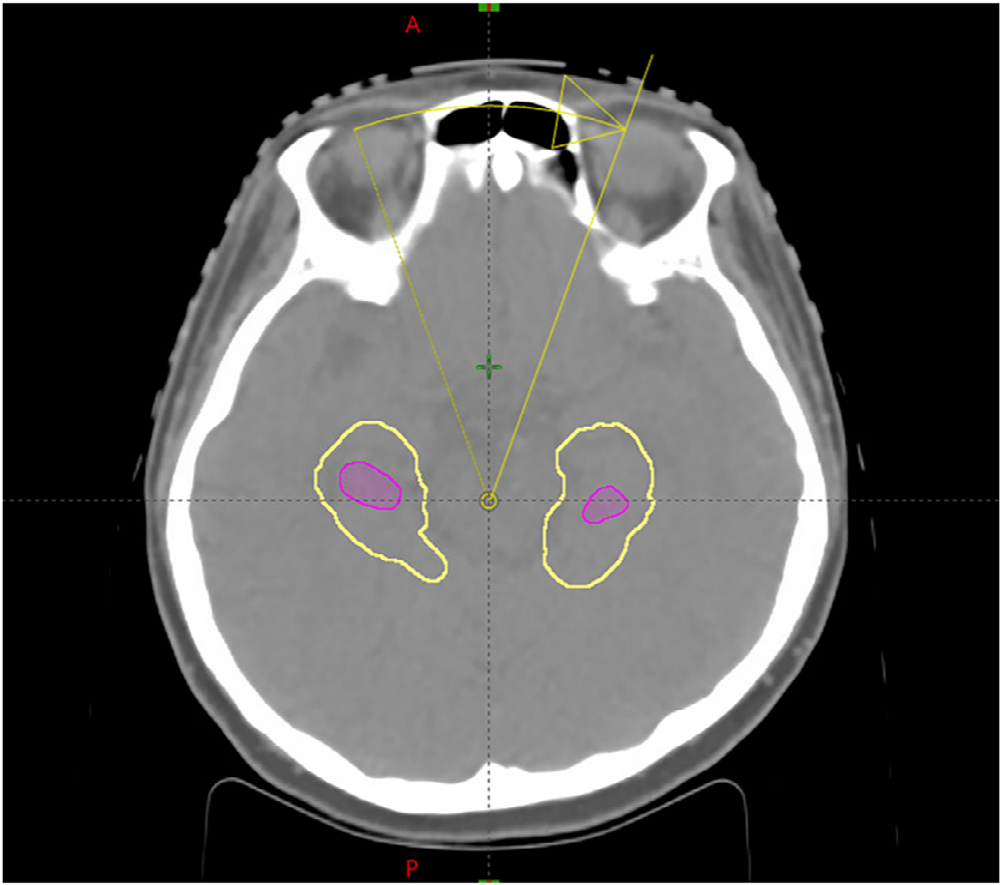
Limited gantry angle from 340° to 20° of coplanar arcs that effectively delivers dose to the portion of whole brain PTV in‐between the hippocampus. The magenta color wash contour is for the hippocampus and the yellow curves are the contour of the hippocampus with 5 mm expansion.

To minimize the hippocampus dose, we tried blocking the hippocampus to both entry and exit beams in the VMAT optimization for 2‐arc coplanar and 4‐arc noncoplanar strategies, however, this method does not work well for the 2‐arc coplanar plans. As seen from Figure 5, only a small 40° gantry angle, ranging from 340° to 20°, can effectively deliver radiation to the whole brain PTV portion that is in‐between the hippocampus. Thus, this portion of the whole brain PTV was underdosed. However, this method works effectively for the 4‐arc noncoplanar plans. For the 2 noncoplanar 180° partial arcs at a couch angle of 90°, radiation can be effectively delivered to the whole brain PTV portion in‐between the hippocampus at all gantry angles. Therefore, due to the hippocampus blocking in VMAT optimization, the 4‐arc noncoplanar method achieves dramatically lower mean and maximum hippocampus dose than the 2‐arc coplanar method, while also achieving whole brain PTV coverage with substantially improved homogeneity.

The phase II randomized study RTOG 0933 was published in 2014. The constraints of the whole brain PTV coverage and hippocampus sparing were partially based on the feasibility of IMRT planning to meet the tolerances. Since then, many researchers investigated whether they could improve upon these. Recently, Yuen et al^22^ achieved a D_100%_ of 7.86 ± 0.08 Gy and D_max_ of 13.23 ± 0.46 for hippocampus, with HI index of 0.23 ± 0.01 for target coverage, using a 4 coplanar‐split‐field‐arcs VMAT planning strategy. Sprowls et al^23^ achieved a mean HI of 0.16 for target coverage, D_100%_ of 8.9 Gy and D_max_ of 15.8 Gy for hippocampus, using HyperArc. Implementation of our planning technique showed dramatically improved hippocampus sparing and target coverage as shown in Table 1. The corresponding indices met the RTOG 0933 dosimetric criteria for both PTV coverage and hippocampus sparing with a great margin. One can also further improve the hippocampus sparing by compromising the target coverage (still meeting the RTOG 0933), or vice versa if needed.

The MUs of the 4‐arc noncoplanar plans was approximately twice those of the 2‐arc coplanar plans. In Sprowls's work, the mean MU for the noncoplanar plans is 681 MU, substantially less than our mean MU of 1790, which is because we pushed hard to minimize the hippocampus dose. Also since our 21ix Linac does not support jaw‐tracking feature, there is a lot more MLC leakage to patients that is not been accounted in isodose, careful IMRT QA is required to ensure the accuracy and clinical acceptability of the plan.

Limited dosimetric dose response relationship has been published from the RTOG 0933 phase II study. Post hoc analysis showed that higher hippocampal D_100%_ predicted for better memory measured by the HVLT‐R DR decline which suggested that further lowering the dose to the entire hippocampus can possibly improve list‐learning recall outcomes.^13^ Further evaluation of potential dose response relationship from RTOG 0933 is currently under investigation. Secondary analysis of RTOG 0933 showed steep dose response relationship between maximal hippocampal dose and memory deficits.^28^ This suggested that by reducing the maximal dose to bilateral hippocampus may preserve cognitive function. Our novel treatment planning technique significantly reduced the D_100%_ D_max_, and D_mean_ for bilateral hippocampus without a cost to coverage of the whole brain PTV.

Although higher biological WBRT dose fractionation did not show a benefit in overall survival (OS), symptom control, or neurological function,^29^ however, underdosing brain metastases can have worse tumor control leading to deterioration of neurological function from intracranial disease progression.^30^, ^31^ Thus, compromising whole brain PTV may possibly translate to worse clinical outcomes. Our current planning technique not only improved hippocampus sparing but also had improved whole brain PTV coverage measured by D_2%_, D_98%_, and HI.

By improving both whole brain PTV coverage and hippocampus sparing, there is a potential to improve the therapeutic ratio of HA‐WBRT. Current ongoing studies are looking to add simultaneous integrated boost (SIB) to HA‐WBRT^32^, ^33^ given that SIB or SRS boost when added to WBRT showed improved local control and intracranial progression free survival.^34^, ^35^ Our current treatment planning technique can be applied to HA‐WBRT SIB treatment planning with minimal adjustments.

## 5 CONCLUSION

For HA‐WBRT, an easy‐to‐implement 4‐arc noncoplanar VMAT planning method is proposed, which uses an intermediate plan while blocking the hippocampus in the optimization. This method dramatically improves the hippocampus sparing and whole brain PTV coverage without compromise to other nearby organs at risk.

## AUTHOR CONTRIBUTIONS

Bei Liu and Ji Kim contributed to the study design, data analysis, and drafting of the manuscript. An Liu and Terence Williams contributed to the data analysis methodology and critical review of the manuscript.

## CONFLICT OF INTEREST STATEMENT

This research has no conflict of interest.
